# Beyond bilateral symmetry: geometric morphometric methods for any type of symmetry

**DOI:** 10.1186/1471-2148-11-280

**Published:** 2011-09-29

**Authors:** Yoland Savriama, Christian Peter Klingenberg

**Affiliations:** 1 Faculty of Life Sciences, University of Manchester, Michael Smith Building, Oxford Road, Manchester M13 9PT, UK

## Abstract

**Background:**

Studies of symmetric structures have made important contributions to evolutionary biology, for example, by using fluctuating asymmetry as a measure of developmental instability or for investigating the mechanisms of morphological integration. Most analyses of symmetry and asymmetry have focused on organisms or parts with bilateral symmetry. This is not the only type of symmetry in biological shapes, however, because a multitude of other types of symmetry exists in plants and animals. For instance, some organisms have two axes of reflection symmetry (biradial symmetry; e.g. many algae, corals and flowers) or rotational symmetry (e.g. sea urchins and many flowers). So far, there is no general method for the shape analysis of these types of symmetry.

**Results:**

We generalize the morphometric methods currently used for the shape analysis of bilaterally symmetric objects so that they can be used for analyzing any type of symmetry. Our framework uses a mathematical definition of symmetry based on the theory of symmetry groups. This approach can be used to divide shape variation into a component of symmetric variation among individuals and one or more components of asymmetry. We illustrate this approach with data from a colonial coral that has ambiguous symmetry and thus can be analyzed in multiple ways. Our results demonstrate that asymmetric variation predominates in this dataset and that its amount depends on the type of symmetry considered in the analysis.

**Conclusions:**

The framework for analyzing symmetry and asymmetry is suitable for studying structures with any type of symmetry in two or three dimensions. Studies of complex symmetries are promising for many contexts in evolutionary biology, such as fluctuating asymmetry, because these structures can potentially provide more information than structures with bilateral symmetry.

## Background

Morphological symmetry results from the repetition of parts in different orientations or positions and is widespread in the body plans of most organisms. For example, the human body is bilaterally symmetric in external appearance because the same anatomical parts are repeated on the left and right sides. Likewise, many flowers are radially symmetric because sets of petals and other organs are repeated in circular patterns. The evolution of morphological symmetry is of interest in its own right [1-8] and variation among repeated parts, such as fluctuating asymmetry, has been widely used for research in evolutionary biology [9-12]. For instance, fluctuating asymmetry can be viewed as a measure of developmental instability [13] and has been related to measures of environmental stress [14], hybridization [15,16], or fitness [17]. In a different context, fluctuating asymmetry can also be used to investigate the developmental origin of morphological integration [11,18-25].

Because bilateral symmetry is the most widespread and simplest type of symmetry, it has been the most studied in various contexts [10]. The symmetry and asymmetry of shape have been studied with the methods of geometric morphometrics [14,16,21-33]. Bookstein [34] and Auffray et al. [26] briefly outlined a procedure for the shape analysis of symmetric structures that are bilaterally symmetric as a whole (e.g. the human skull). Mardia et al. [35] and Kent and Mardia [36] established the mathematical basis and computational procedure for this method, and Klingenberg et al. [31] integrated it with the existing methods for the study of biological symmetry and asymmetry.

**Figure 1:**
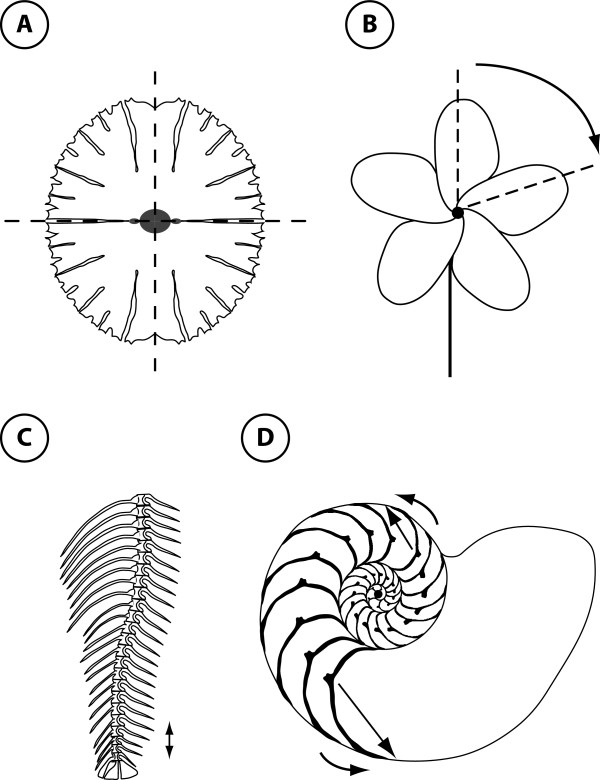
**Some types of symmetry found in the structure of living organisms**. A. An alga (*Micrasterias rotata*) with two perpendicular axes of reflection symmetry (dashed lines; biradial symmetry or disymmetry). B. A flower, (*Plumeria alba*) that shows rotational symmetry (the axis of rotation is shown by the black dot at the centre of the flower and the angle of rotation is represented by the curved arrow). C. The arrangement of vertebrae of a zebrafish exhibits translational symmetry (the translation is indicated by the double-headed arrow). D. A cross-section of a nautilus shell (*Nautilus pompilius*) showing scale symmetry that is a combination of rotations, translations and dilations (the centre of rotation is represented by the black dot, the rotation by the curved arrow, and the translation by the straight arrow).

Whereas bilateral symmetry has been the focus of most studies, it is not the only kind of symmetry in living organisms (Figure 1). A range of different types of symmetry occur both in plants and animals [2,3,12]. So far, there is no general method for the morphometric analysis of all these types of symmetry, although some methods have been proposed for specific types [37,38] and an application of the present methodology to one type of symmetry has been published [39]. In other contexts, however, related approaches have been developed: for instance, in chemistry and computer vision a general method for quantifying how close a single configuration of points is to a symmetric shape, for any type of symmetry [40,41], and in engineering a method to incorporate symmetry in computations of the mechanical loading of structures [42]. No general framework exists, however, that extends the existing concepts and methods for studying morphometric variation of symmetric structures and biological asymmetry to all types of symmetry.

In this paper, we generalize the approach of Mardia et al. [35], which is restricted to bilateral symmetry, for the shape analysis of structures with any type of symmetry. Our generalization is based on the theory of symmetry groups [43-47], which is long established in mathematics, but so far has not been used in the context of morphometric shape analysis. We focus on symmetric structures that possess anatomical landmarks, and thus exclude smooth symmetric structures such as circles, spheres, or ellipsoids because landmarks cannot be located on them. Our method can separate a component of symmetric variation among individuals from one or more asymmetry components, depending on the specific type of complex symmetry.

This paper first reviews the mathematical definition of symmetry and the theory of symmetry groups [43-47], which may not be familiar to evolutionary biologists. We show that the approach of Mardia et al. [35] for bilateral symmetry is a special case of a more general framework based on symmetry groups. We explain how the concepts of fluctuating and directional asymmetry can be extended to all types of symmetry. We also generalize Procrustes ANOVA, a method for quantifying symmetric and asymmetric components of shape variation [29,31], to complex types of symmetry. Finally, we illustrate this new approach with an example using landmark data collected from skeletal structures of a colonial coral. Because the symmetry of these structures is ambiguous, we are able to demonstrate analyses with different types of symmetry for this data set.

## Results

### Mathematical definition of symmetry

Many types of symmetry exist in nature besides the familiar bilateral symmetry. Some organisms, such as the green alga *Micrasterias *(Figure 1A) have two perpendicular axes (or planes) of reflection symmetry (also called biradial symmetry or disymmetry). Others exhibit rotational symmetry (or radial symmetry) where parts are arranged around a central point (or axis) so that each part is rotated from the neighbouring ones by a certain angle (Figure 1B). Many organisms show translational symmetry, better known as serial homology, in which the body is divided into a suite of parts that are repeated along the body axis (Figure 1C). Many mollusc shells (e.g. snails, nautilus, and ammonites) show scale symmetry (or spiral symmetry): rotational symmetry is combined with translational symmetry and dilation, so that the object gradually expands from one whorl to the next (Figure 1D). These basic types of symmetry can be combined to produce a large diversity of symmetry patterns. Unfortunately, this biological conception of symmetry is not suitable for a rigorous quantitative study of symmetry.

Instead, we use a definition of symmetry that was developed in a mathematical context [43-47]. To derive the definition, we consider the changes that an object undergoes when a geometric transformation is applied to it. A transformation is a geometric change that maintains a one-to-one correspondence among all points of the object in the plane (or space) [45]. In other words, a transformation maps every point of the object in the plane (or space) onto another point in the same plane (or space) and is reversible (it is possible to map back from the second to the first point). D'Arcy Thompson's [48] transformation grids are a visualization of transformations in two dimensions, which are familiar to most morphometricians. In the context of symmetry, some particular transformations are of special interest. The simplest of these transformations is the identity that maps every point of the plane onto itself. Other transformations important for the study of symmetry are translation (a shift of the object in some direction), rotation, reflection, stretch (magnifying or shrinking), and their combinations [45]. Usually, applying a transformation to an object changes the object so that at least some of its points are in different positions before and after the transformation.

**Figure 2:**
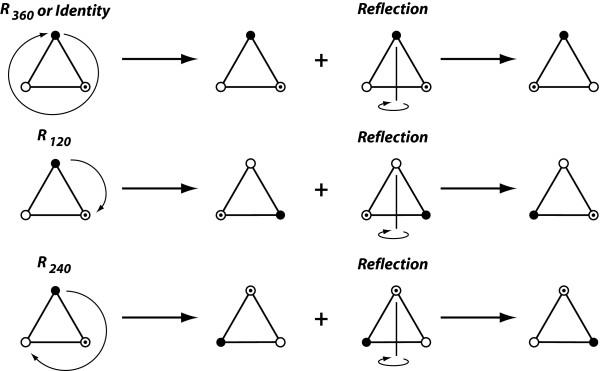
**The set of symmetry transformations that define the symmetry group of the equilateral triangle**. This symmetry group includes six symmetry transformations: the identity, rotations of order 3, and combinations of reflection with rotations.

In geometry, symmetry is defined as invariance of an object to a particular transformation that can be applied to it, such that the object is the same before and after transformation [43-46]. The object remains unchanged after the transformation precisely if it is symmetric with respect to that transformation. For instance, the human face is symmetric because it is invariant to reflection about the median plane. This transformation brings the left side of the face onto the right side and vice-versa, but because the two sides are mirror images of each other, the face as a whole is left unchanged (up to minor asymmetries). Objects can have several symmetries if they are invariant to several transformations. For instance, the equilateral triangle is invariant to rotation by 120°, rotation by 240° and to reflection about three axes (or equivalently, a reflection about one axis combined with the rotations; Figure 2). In addition, every object is symmetric with respect to the transformation that does not change the object, which is called the identity (and is equivalent to a rotation by 360° or two successive reflections about the same axis or plane). The transformations that leave an object unchanged are called the symmetry transformations for that object. Which transformations are symmetry transformations depends on the object: there are many objects that have no symmetry at all and therefore have no symmetry transformations (other than the identity), whereas other objects may have several.

The symmetry transformations of an object jointly characterize its symmetry. For instance, bilateral symmetry is defined by two symmetry transformations: reflection and the identity. The set of symmetry transformations of an object forms a special kind of set called a symmetry group [e.g. [44-46]]. The symmetry group has four special properties, as follows. (1) If any two symmetry transformations are combined, the result is another transformation that is contained in the symmetry group (closure of the group). (2) The symmetry group contains the identity as one of its elements. (3) For every symmetry transformation, there is a transformation in the symmetry group that undoes the change produced by the first transformation (each element of a group has an inverse). The inverse may be the symmetry transformation itself (e.g. applying a reflection about the same axis twice results in no change at all). Finally, (4) for combinations of any three symmetry transformations *a*, *b *and *c*, the equality (*ab*)*c *= *a*(*bc*) holds (associativity). Due to these properties, the powerful mathematical tools of group theory can be used for studying symmetry [e.g. [44-46]]. For bilateral symmetry, the symmetry group contains just two transformations: the identity and the reflection about the axis or plane of symmetry for the object. For the symmetry of the equilateral triangle, the symmetry group contains six elements combining rotations (by 120°, 240° and 360° or 0°) and a reflection (Figure 2).

The order of a group is the number of distinct elements in the group. Some symmetry groups are of finite order, because the repeated application of the same transformation eventually maps the object onto itself and thus produces only a finite number of different transformations (e.g. a rotation by 120° can only be applied three times to yield the rotations by 120°, 240° and 360° or 0°). These are called finite symmetry groups. All finite symmetry groups in two and three dimensions consist of rotations and reflections (and their combinations; e.g. Figure 1A, B) [46]. In contrast, any symmetry groups that contain translations are not finite, because for any transformation that consists of applying a translation *n *times, the symmetry group must also contain the transformation that consists in applying the translation *n *+ 1 times, and so on. Similarly, if a transformation changes the scale of the object, repeated application of this transformation makes the object either larger and larger or smaller and smaller without end. Therefore, translational symmetry (Figure 1C) and spiral symmetry (Figure 1D) are associated with infinite symmetry groups.

### Matching symmetry and object symmetry

In their paper on shape analysis of bilateral symmetry, Mardia et al. [35] distinguished matching symmetry, where there are pairs of separate structures on the left and right sides, from object symmetry, where a single structure is internally symmetric. Matching symmetry concerns pairs of structures that are present as physically separated mirror images on the left and right sides (e.g. the human hands). The analysis uses the separate landmark configurations for the left and right structures of each individual in the sample, applies a reflection to the configurations from one side, and then a Procrustes fit to the combined sample [29]. Matching symmetry can be extended to other types of symmetry in a straightforward manner: instead of pairs of structures on the left and right sides, there is simply a different arrangement of multiple corresponding parts that reflects the symmetry in question. Examples of matching symmetry are found in the petals of radially symmetric flowers (Figure 1B) or the vertebrae that make up the translational symmetry of the vertebral column (Figure 1C). Analyses of matching symmetry focus on the landmark configurations for the repeated parts, rather than on the composite structure as a whole. A first step in the analysis is therefore to divide a structure into parts (e.g. a flower into separate petals), and corresponding landmarks are then recorded on each of the parts. Because this decomposition into parts is possible regardless of the structure or order of the symmetry group, analysis of matching symmetry is possible for any type of symmetry.

Object symmetry concerns structures that are symmetric as a whole [31,35]. For instance, the vertebrate skull has object symmetry because a plane of bilateral symmetry passes through the middle of the skull, and the skull as a whole is invariant under reflections about this plane. Structures with other types of symmetry can also be invariant as a whole under the symmetry transformations of their respective symmetry group. For instance, the algal cell in Figure 1A is invariant under reflections about the horizontal and vertical axes (or, equivalently, under one reflection and a rotation by 180°) and the flower in Figure 1B is invariant as a whole under five rotations. As for the case of bilateral symmetry, the axes or planes of reflection symmetry pass through the configuration of landmarks and the centre or one or more axes of rotation are situated inside the configuration.

Because object symmetry requires the entire object to be invariant under the transformations in the symmetry groups, some limitations are imposed on the types of symmetry for which object symmetry is possible. For instance, an object cannot be invariant to transformations involving a change of scale. Likewise, a finite object cannot be invariant to transformations involving translations because at least the ends of the object would not be invariant. Moreover, for objects with distinct landmarks (i.e. excluding circles and spheres without any identifiable landmarks), rotations are limited to those by an angle of 360°/*n *(where *n *is an integer ≥ 1) and multiples thereof. As a consequence of these limitations, object symmetry applies only to those types of symmetry that are associated with finite symmetry groups. These symmetry groups consist of rotations, reflection, or a combination of rotations and reflections [e.g. [43,45,46]]. For types of symmetry associated with infinite symmetry groups, such as translational or spiral symmetry (Figure 1C, D), shape analysis must use matching symmetry by focusing on the repeated parts, rather than the structure as a whole.

### Shape analysis of matching symmetry

#### Bilateral symmetry

For studies of structures with matching symmetry, the analysis starts with a pair of separate landmark configurations from the left and right sides of each individual in the sample. First, the landmark configurations from one side are reflected. Then, a generalized Procrustes fit superimposes all configurations and produces an overall mean shape [e.g. [28,29]]. The Procrustes fit, by definition, automatically adjusts for rotations and translations of the configurations to bring all of them into a standard position and orientation [e.g. [49]].

The variation around the consensus is decomposed into a component of variation among individuals and a component of left-right asymmetry. The variation among individuals is computed from the average shapes of the left and right sides for each individual in the sample. Asymmetry is quantified from the shape differences between the left and right sides for each individual. The average asymmetry is interpreted as directional asymmetry and the individual variation in asymmetry as fluctuating asymmetry [for details see [29,31]].

To quantify the different effects, we can use the decomposition of the sum of squared Procrustes distances of the configurations from the overall mean shape [26,28,29,50]:

(1) $$\overset{n}{\underset{i=1}{\sum }}\underset{s=\text{L,R}}{\sum }D^{2}\left(x_{is},\bar{x}\right)\;=\;2\overset{n}{\underset{i=1}{\sum }}D^{2}\left({\bar{x}}_{i\cdot },\bar{x}\right)\;\;\;+\;\;n\underset{s=\text{L,R}}{\sum }D^{2}\left({\bar{x}}_{\cdot s},\bar{x}\right)\;+\;\overset{n}{\underset{i=1}{\sum }}\underset{s=\text{L,R}}{\sum }D^{2}\left(x_{is},{\bar{x}}_{i\cdot }+{\bar{x}}_{\cdot s}-\bar{x}\right)$$

In this equation, the expression *D*^2^(**a**, **b**) denotes the squared Procrustes (tangent) distance between two landmark configurations **a **and **b **(landmark configurations are written as vectors of coordinates after Procrustes superimposition and projection to tangent space). The landmark configuration **x***_is _*is the configuration for side *s *of the *i*-th individual (where the subscript *s *can take the values L and R for the left and right side, respectively). The overall mean configuration is $\bar{x}$, the consensus of the *i*-th individual is ${\bar{x}}_{i\cdot }$ and ${\bar{x}}_{\cdot s}$ stands for the mean for side *s *across individuals (i.e. the mean of all left or right sides). The first sum on the right-hand side of the equation stands for the shape variation among individuals, the second sum stands for directional asymmetry, and the third sum stands for fluctuating asymmetry. This decomposition of the total sum of squares corresponds to the two-factor ANOVA design of Leamy [51] and Palmer and Strobeck [52], and it is the basis for the Procrustes ANOVA [26,29,31].

Note that the decomposition of the sum of squared Procrustes distances and Procrustes ANOVA usually are only preliminary steps to quantify the relative amounts of variation at the different levels. In particular, reducing shape variation to a scalar distance measure ignores the patterning of variation in the multidimensional space. To extract information from those patterns, the preliminary assessments based on Procrustes distances are usually followed by multivariate analyses of individual variation and asymmetry that can address a wide range of biological questions [11,18,21,24,30,33,53-56]. Depending on the particular questions of interest, methods used in these analyses can include principal component analysis [29,33,57], partial least squares [21,57,58], matrix correlation [29,33,53], or tests of modularity [20].

#### Other types of symmetry

Matching symmetry can be generalized directly from bilateral symmetry to any type of symmetry. The main difference is the number and arrangement of the parts that are considered for each individual in the sample, which also results in different components of asymmetry. Instead of a pair of parts on the left and right sides, there may be more than two parts, which can be arranged in many different ways. For example, the flower of Figure 1B has five petals, which can be included in the analysis as five separate configurations of landmarks. Depending on the arrangement of parts, reflection may need to be used to match corresponding landmarks for all configurations. Finally, a Procrustes fit is used to superimpose all configurations simultaneously and components of variation around the consensus shape can be extracted.

The partitioning of the components of variation extends the type of analysis used for bilateral symmetry. A component of variation among individuals is computed from the averages of the landmark configurations of parts for each individual. Directional asymmetry can be visualised by comparing the mean shape for each of the repeated parts to the grand mean across all parts. Fluctuating asymmetry is the individual variation of the deviations of each repeated part from the individual average of all parts.

The decomposition of the sum of squared Procrustes distances of the shapes of the landmark configurations from the overall mean shape is also a direct extension of the formula for bilateral symmetry (equation 1):

(2) $$\overset{n}{\underset{i=1}{\sum }}\overset{p}{\underset{s=1}{\sum }}D^{2}\left(x_{is},\bar{x}\right)\;=\;p\overset{n}{\underset{i=1}{\sum }}D^{2}\left({\bar{x}}_{i\cdot },\bar{x}\right)\;+\;n\overset{p}{\underset{s=1}{\sum }}D^{2}\left({\bar{x}}_{\cdot s},\bar{x}\right)\;+\;\overset{n}{\underset{i=1}{\sum }}\overset{p}{\underset{s=1}{\sum }}D^{2}\;\left(x_{is},{\bar{x}}_{i\cdot }+{\bar{x}}_{\cdot s}-\bar{x}\right)$$

In this equation, the summation over the parts for each individual is not limited to the left and right sides, but can accommodate any number of repeated parts, *p*, for each individual (the subscript *s *can take any value from 1 to *p*). Otherwise, the decomposition is identical to equation (1) and it also yields three separate sums of squared Procrustes distances that correspond to the variation among individuals, directional and fluctuating asymmetry.

The decomposition in equation (2) is a "minimal" version in which the asymmetry, the variation among parts of each individual, is included as a single component (contributing to both fluctuating and directional asymmetry). For example, an analysis of variation in a sample of radially symmetric flowers can use the variation among petal shapes to extract a component of directional asymmetry (deviation of the mean shape of petals at each position from the overall mean of all petals) and a component of fluctuating asymmetry (individual deviations from the mean shape of the respective petal).

Depending on the number and arrangement of parts, however, it may be possible to decompose asymmetry further into multiple components. Such a more complex decomposition is possible for the example of the alga in Figure 1A, because its symmetry results from a reflection about the horizontal axis and a rotation by 180° (or, equivalently, another reflection about the vertical axis) [39]. Accordingly, two separate components of asymmetry can be distinguished: asymmetry in the vertical and horizontal directions (in other organisms, these might correspond to dorsoventral and lateral differences, respectively) and an "interaction" effect that indicates how much each of the four "quadrants" differs from the combined effects of the two main asymmetries. This partitioning applies both to the mean asymmetries (directional asymmetry) and to the individual variation of asymmetries (fluctuating asymmetry). Depending on the arrangement of repeated parts, the information gained from the analysis of symmetry and asymmetry therefore may be substantially more intricate than for bilateral symmetry.

As in the case of bilateral symmetry, a variety of multivariate analyses can be used to investigate the patterns of variation in the differences or averages among repeated parts. This is a potentially rich, but so far unexplored area for morphometric studies.

#### Parts with object symmetry

For many structures that have matching symmetry overall, the repeated parts themselves show bilateral object symmetry. This applies to the vertebrae in a vertebral column (Figure 1C) or the chambers in a *Nautilus *shell (Figure 1D), as well as for the petals of many flowers (although not for the example in Figure 1B). In this case, the object symmetry of the parts can be combined with the data structure that reflects the overall matching symmetry. For instance, an analysis of the vertebral column may include the anterior-posterior sequence of vertebrae and the bilateral object symmetry of each vertebra [31]. Such an analysis would include the original and reflected and relabelled copies of each measured vertebra in the vertebral column to take into account the object symmetry of each vertebra. An analysis of anterior-posterior differences among vertebrae would use the left-right symmetric component computed from the averages of the original and reflected copies of each vertebra. Analyses of asymmetry might be conducted separately for each vertebra, or they might be done in combination to examine changes in asymmetry along the vertebral column (e.g. changes in directional asymmetry).

### Shape analysis of object symmetry

#### Bilateral symmetry

Because the shape analysis for object symmetry is somewhat more complex than for matching symmetry, we review the established method for analyzing bilateral object symmetry [31,35,36] and provide a new explanation from the perspective of symmetry groups, which we then use as the starting point for the generalization to any type of symmetry. The analysis of left-right variation with object symmetry is based on a single configuration of landmarks that includes both sides of the structure. There are two types of landmarks in the original configuration: paired and unpaired landmarks [31,35,36]. The paired landmarks occur on each side of the structure outside the middle plane or axis (e.g. the corners of the mouth), whereas unpaired landmarks are located in the middle plane or axis (e.g. the tip of the nose).

For each specimen, a reflected copy of the original configuration of landmarks is produced (e.g. by changing the signs for one of the coordinates of all landmarks). Because the reflection brings the landmarks from the right side onto the left side and vice versa, the paired landmarks are relabelled by exchanging the labels between left and right paired landmarks of the reflected copy to make them compatible with the paired landmarks of the original configuration. Finally, a Procrustes fit superimposes both the original configurations of landmarks and their reflected and relabelled copies [31,35,36].

The resulting consensus configuration is symmetric under reflection [31,35,36]. Because a reflected and an unchanged copy of the original configuration are used for each individual, applying reflection and relabelling to all configurations will yield the same pair of configurations (the original will be mapped to the reflected and relabelled copy, and vice versa), and the Procrustes fit will produce the same average shape. Therefore, the pair of configurations used for each specimen and the resulting average shape are invariant under reflection and relabelling, and thus the average shape is symmetric [[35], p. 287].

The variation around the consensus is decomposed into a component of symmetric variation and a component of asymmetry [31,35,36]. The symmetric component is computed from the average of the original configuration and reflected and relabelled copy of each individual. Symmetric variation is the variation of these averages among the individuals in the data. Asymmetry is calculated from the differences between the original configurations and reflected and relabelled copies. Directional asymmetry is the average asymmetry, whereas fluctuating asymmetry is the variation of the individual asymmetries [31,35,36].

Mardia et al. [[35], p. 288] provided a decomposition of the sum of squared Procrustes distances between the original configurations and the corresponding reflected and relabelled copies. We can expand this slightly, in line with equations (1) and (2), by providing the complete decomposition of the sum of squared Procrustes distances of each configuration from the overall consensus:

(3) $$\overset{n}{\underset{i=1}{\sum }}\underset{s=\text{O,R}}{\sum }D^{2}\left(x_{is},\bar{x}\right)\;=\;2\overset{n}{\underset{i=1}{\sum }}D^{2}\left({\bar{x}}_{i\cdot },\bar{x}\right)\;\;\;+\;\;n\underset{s=\text{O,R}}{\sum }D^{2}\left({\bar{x}}_{\cdot s},\bar{x}\right)\;+\;\overset{n}{\underset{i=1}{\sum }}\underset{s=\text{O,R}}{\sum }D^{2}\left(x_{is},{\bar{x}}_{i\cdot }+{\bar{x}}_{\cdot s}-\bar{x}\right)$$

Note that this expression is identical to the one for bilateral matching symmetry (equation 1) except for the subscript *s*, which now takes the values O and R for the original and reflected copy, respectively.

The patterns of covariation in the symmetric and symmetric components of variation can be further explored in multivariate analyses to answer a wide range of questions. Such analyses can use principal component analysis [22,31,59], partial least squares [25], matrix correlation [22,31], multivariate regression [22,60,61], or modularity tests [22,23,25,59,62].

It is useful at this point to reconsider this established method from the new perspective of symmetry groups. For bilateral symmetry, the symmetry group consists of just two symmetry transformations: the identity and a reflection. The unchanged and reflected copies of each configuration that are included in the Procrustes fit can therefore be interpreted as a set of copies of each original configuration to which the whole set of symmetry transformations has been applied. The relabelling serves to map each landmark to the corresponding one after the transformation: for the reflection, each paired landmark is mapped to its equivalent on the opposite side and each median landmark is mapped to itself (for the identity, every landmark is mapped to itself).

This reasoning can also be used to understand why the Procrustes average of the unchanged copy and the copy that has been reflected and relabelled must be symmetric. The pair of unchanged and reflected copies of each configuration is invariant under reflection because the two transformations that were applied to the original configuration (identity and reflection) constitute the entire symmetry group. If we combine any given symmetry transformation with each of the transformations in the symmetry group, the resulting transformations must also be elements of the symmetry group. Therefore, the set of all the resulting transformations is the same as the original symmetry group. As a consequence, the Procrustes average of the complete set of copies must be the same before and after the transformation, and therefore is symmetric.

These explanations from the perspective of symmetry groups may seem an overly complicated manner of describing the procedure and of justifying why the consensus is symmetric, but this new reasoning has the advantage of offering a direct way to generalize the analysis to more complex types of symmetry.

#### Generalization to other types of symmetry

Using the reasoning based on symmetry groups, it is possible to extend the method for analyzing object symmetry to any type of symmetry that is associated with a finite symmetry group. All finite symmetry groups can be generated by reflection, rotation or a combination of both. For configurations in two dimensions, a symmetry group can contain only a single rotation about a central point. This rotation can have different orders, the number of repeated steps it takes to cover a full circle (i.e. for a rotation of order *o*, each step is a rotation by 360°/*o*). Overall, therefore, reflection, rotation of different orders, and their combinations are the only kinds of object symmetry in two-dimensional data. In three dimensions, however, there can be rotations about a single axis, about two perpendicular axes, or the more complex arrangement of axes in the symmetries of the platonic solids (tetrahedron, cube/octahedron, dodecahedron/icosahedron). In total, there are 14 types of finite symmetry groups in three-dimensional space [43-47], which are listed in Table 1. Of these, the first two symmetry groups, consisting of rotations about a single axis with or without reflection, are by far the most frequent in biological structures, whereas most other types are rarely found (although radiolarians are famous for a wide range of symmetries, including those of the Platonic solids [e.g. [43]]).

**Table 1:** Enumeration of all finite symmetry groups in 3D space, with the Schoenflies and orbifold notations and the order of each group [47]

Schoenflies	Orbifold	Order	Comments
C_*n*_	*nn*	*n*	Rotational symmetry of order *n*
C_*n*v_	*nn	2*n*	Rotational symmetry of order *n *and reflection symmetry about *n *planes containing the rotation axis
C_*n*h_	n*	2*n*	Rotational symmetry of order *n *and reflection symmetry about a plane perpendicular to the rotation axis
S_2*n*_	*n*×	2*n*	Rotational symmetry of order 2*n *in which odd-numbered elements are reflected about a plane perpendicular to the rotation axis
D_*n*_	22*n*	2*n*	Dihedral symmetry: rotational symmetry of order *n *combined with rotational symmetry of order 2 about axes that are perpendicular to the first rotation axis
D_*n*d_	2*n	2*n*	Antiprismatic symmetry: Rotation symmetry of order *n *and reflection symmetry about *n *planes containing the rotation axis, as well as rotation symmetry of order 2 about a perpendicular axis in each of the resulting sectors
D_*n*h_	*22*n*	4*n*	Prismatic symmetry: rotational symmetry of order *n *and reflection symmetries about planes *n *passing through the rotation axis as well as the plane perpendicular to it.
T	332	12	Tetrahedral symmetry, rotations only
T_d_	*332	24	Complete tetrahedral symmetry, including reflection
T_h_	3*2	24	Pyritohedral symmetry
O	432	24	Octahedral symmetry, rotations only (also applies to cube)
O_h_	*432	48	Complete octahedral symmetry, icluding reflection (also applies to cube)
I	532	60	Icosahedral symmetry, rotations only
I_h_	*532	120	Complete icosahedral symmetry, including reflection

Bilateral symmetry can be viewed as a special case of C_*nv *_or C_*nh *_with a rotation of order 1.

The idea for shape analysis with object symmetry of any type is to assemble a data set containing copies of each original landmark configuration to which all the transformations in the symmetry group have been applied with the appropriate relabelling, and then to perform a generalized Procrustes fit on this data set. As for bilateral symmetry, the resulting consensus shape is symmetric and it is possible to extract components of symmetric and asymmetric variation by computing the appropriate averages or differences between landmark configurations after the Procrustes fit.

Because all finite symmetry groups consist of rotations and reflections, it is sufficient to consider the steps to produce the transformed and relabelled copies for reflection and rotation. For reflection, there are paired and unpaired landmarks. The reflection itself can be carried out by changing the sign of one coordinate for all landmarks (e.g. all *x *coordinates). The relabelling maps each paired landmark to the corresponding landmark of the opposite side and each unpaired landmark to itself. For rotations, there are two types of landmarks: landmarks on the centre or axis of rotation, and the remaining landmarks, which are repeated in each of the sectors defined by the rotation. To prepare rotated copies of the original configuration for the combined dataset, only the relabelling is necessary, because the Procrustes fit will automatically perform the appropriate rotations to fit the relabelled landmark configurations to each other. A separate relabelled copy is produced for rotation by one step, two steps, and so on up to the order of the rotation (e.g. for a rotation of order 4, there would be four copies with rotations by 90°, 180°, 270°, and 360°, i.e. including the identity). It does not matter whether the rotations are done clockwise or counter-clockwise, because all possible steps are included in either way. If the symmetry group contains reflection and rotation or more than one rotation, all possible combinations need to be included in the combined dataset. For instance, if an object has rotational symmetry of order three and reflection symmetry (the symmetry group of the equilateral triangle, Figure 2; e.g. the *Iris *flower), six copies of each original landmark configuration will be included in the combined data set.

The generalized Procrustes fit of the combined data set produces a consensus shape that is invariant under all the transformations in the symmetry group, that is, a completely symmetric shape. Because the set of transformations used to produce the combined dataset is the complete symmetry group, applying any of the symmetry transformations leaves the set unchanged as a whole, and therefore does not alter the average shape resulting from the Procrustes fit. Therefore, the consensus shape is invariant under all the symmetry transformations, and is thus perfectly symmetric. This is true both for the consensus shapes for individual specimens (the Procrustes consensus for the set of the original and transformed copies of just one specimen at a time) and for the combined Procrustes fit of all specimens jointly (all copies for all individuals).

**Figure 3:**
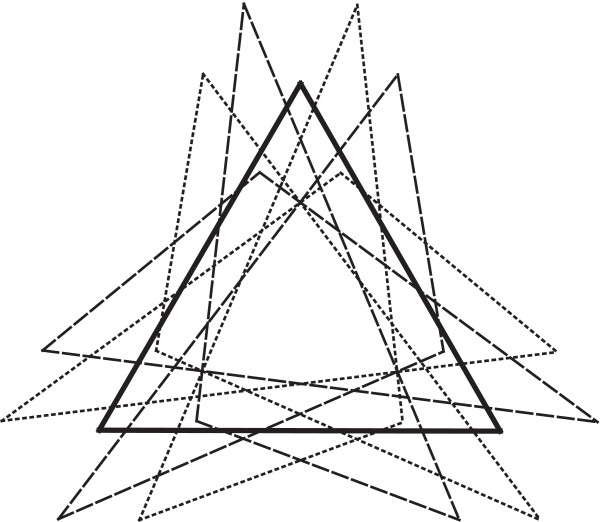
**The Procrustes fit of the transformed and relabelled copies of a single triangle to the symmetric consensus**. The diagram shows the symmetric mean shape (bold solid triangle) and six copies of the triangle that have been transformed and relabelled using six symmetry transformations: the identity, rotations of order 3, and combinations of reflection with rotations (i.e. this is the same symmetry group as in Figure 2). Copies of the triangle for which the transformation does or does not include reflection about the vertical axis are distinguished by dashed and dotted lines.

The final alignment of landmark configurations in the generalized Procrustes fit is obtained by an ordinary Procrustes fit of each configuration to the consensus shape [49]. Because the consensus shape is perfectly symmetric under all transformations in the symmetry group, the alignments of the transformed and relabelled copies of each configuration all share the same centroid and differ exactly by the symmetry transformations. For instance, the six transformed copies of the triangle in Figure 3 differ from each other by rotations of exactly 120° or 240°, by a reflection about the vertical axis, or a combination of both (each with the appropriate relabelling).

Because of the symmetry of the consensus configuration, the Procrustes distance between it and every transformed and relabelled copy must be the same for each specimen included in the analysis. This applies both for the consensus configuration for each particular specimen and for the grand mean across all copies of all specimens included in an analysis (and the distance to the particular specimen's consensus shape is less than or equal to the distance to the overall consensus shape). These regularities have direct consequences for the structure of variation in the shape tangent space. Recall that usually the mean shape is chosen as the tangent point [49], the point where the tangent space touches Kendall's shape space and which also serves as the origin for the coordinate system in the tangent space; in the present context, this is the completely symmetric consensus. Because all copies of each specimen have the same Procrustes distance from that specimen's consensus and all copies have the same distance from the overall consensus, these copies are located on a hypersphere in shape tangent space (the intersection between two hyperspheres, one around the overall consensus and one around that specimen's consensus, with radii equal to the respective shape distances). Moreover, copies differing by a rotation of a given angle about the same centre or axis are separated by equal Procrustes distances (e.g. for a rotation of order 6, pairs of copies differing by a rotation of 60° are separated from each other by the same Procrustes distance; likewise, copies differing by a rotation of 120° are separated by identical Procrustes distances and thus form an equilateral triangle in shape tangent space).

It is possible to extract and quantify components of symmetric and asymmetric variation by averaging the transformed and relabelled copies or by computing differences between them. The decomposition of the total sum of squared Procrustes distances in a sample into these components of symmetric and asymmetric variation is a direct extension of the decomposition for bilateral symmetry (equation 3):

(4) $$\overset{n}{\underset{i=1}{\sum }}\overset{o}{\underset{s=1}{\sum }}D^{2}\left(x_{is},\bar{x}\right)\;=\;o\overset{n}{\underset{i=1}{\sum }}D^{2}\left({\bar{x}}_{i\cdot },\bar{x}\right)\;+\;n\overset{o}{\underset{s=1}{\sum }}D^{2}\left({\bar{x}}_{\cdot s},\bar{x}\right)\;+\;\overset{n}{\underset{i=1}{\sum }}\overset{o}{\underset{s=1}{\sum }}D^{2}\;\left(x_{is},{\bar{x}}_{i\cdot }+{\bar{x}}_{\cdot s}-\bar{x}\right)$$

The difference to equation (3) for bilateral symmetry is that any symmetry group can be accommodated. Accordingly, the subscript *s*, which stands for the symmetry transformation, now runs from one to *o*, the order of the symmetry group (i.e. **x***_is _*is the copy of the *i*-th configuration to which the *s*-th transformation in the symmetry group has been applied). The sums over all transformation in the symmetry group may have more than two summands (if *o *> 2). As for bilateral symmetry, this decomposition provides separate sums of squares for the symmetric component of variation and for directional and fluctuating asymmetry.

A component of symmetric variation among individuals can be computed from the variation among the average shapes for the complete set of copies for each specimen. Directional asymmetry can be obtained as the difference of the average shape of all original configurations from the overall Procrustes consensus of the original and transformed copies. Fluctuating asymmetry can be computed from the variation of the individual asymmetries. These computations are direct extensions of those for bilateral object symmetry [31,35]. The difference is that there may be more than one component of asymmetry, depending on the transformations included in the symmetry group. In this case, some of these components of asymmetry will be partially symmetric (symmetric under some of the transformations in the symmetry group, but not under others). For instance, for the symmetry group of rotations of order 4, there is an asymmetry component that is symmetric under rotations of order 2 but asymmetric under rotations of order 4, in addition to a component asymmetric under rotations of any order.

The symmetric and asymmetric components of variation occupy mutually orthogonal subspaces of the shape tangent space, again extending the situation found for bilateral symmetry [31,35,36]. These subspaces can be characterized and their dimensionality can be obtained by enumerating the degrees of freedom [31,35]. Because the subspaces differ among symmetry groups, this must be done separately for each type of symmetry. For this purpose, it is helpful to consider the orbifold [47] for the symmetry group of interest, the portion of the configuration that is repeated by the symmetry transformations, and the landmarks contained in it and on its borders (for further information on orbifolds, see [47]).

Here, we only work out the dimensionalities of these subspaces for symmetry groups containing a single rotation with or without reflection. Together with bilateral symmetry, these are all the possible types of complex object symmetry for two-dimensional data (i.e. all finite symmetry groups in 2D). For three-dimensional data, however, these two types do not cover all possibilities for complex object symmetry (cf. Table 1, groups C*_n _*and C_*nv*_), but they are by far the most widespread types of complex symmetry in the shapes of organisms and their parts (radial and disymmetric floral symmetries, the body plans of cnidarians and echinoderms, diatom cells, etc.).

**Figure 4:**
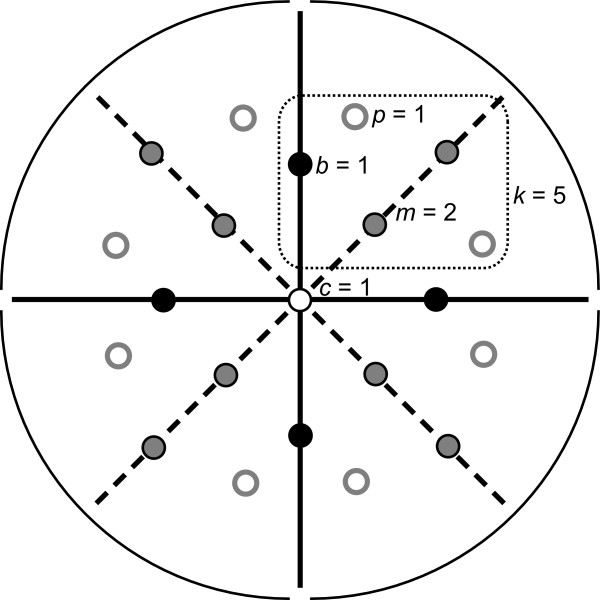
**Sectors and types of landmarks for complex object symmetry with a rotation and reflection**. To compute the dimensionalities of the different components of shape space, it is helpful to subdivide the configuration of landmarks into sectors and to distinguish different types of landmarks. The diagram shows an example of symmetry under rotation of order 4 and reflection. Therefore, the configuration can be divided into four sectors: the regions that correspond to each other when the rotation is applied (sector boundaries are indicated by solid black lines). If the symmetry also includes reflection, as in this example, the arrangement of landmark in each sector is also bilaterally symmetric about the midline or mid-plane of each sector (dashed lines). Several types of landmarks can be distinguished. There may be a landmark in the centre of rotation or, for 3D data, there may be multiple landmarks of the axis of rotation (*c *= 0, 1 for 2D data; *c *≥ 0 for 3D data). Each sector contains *k *landmarks. If the order of rotation is denoted *o*, the total number of landmarks is therefore *c *+ *ko *(in the diagram, *c *= 1, *k *= 5 and *o *= 4, so that there are 1 + 5 × 4 = 21 landmarks). If the symmetry group contains reflection as well as a rotation, the *k *landmarks of each sector can be subdivided into *b *landmarks on the sector boundary, *m *landmarks on the midline or mid-plane of the sector, and *p *pairs of corresponding landmarks on either side of the midline (therefore, *k *= *b *+ *m *+ 2*p*). We define the sector boundary as running through the axis or plane of reflection on at least one side of the centre or axis of rotation (if the order of rotation is even, two sector boundaries are in the axis or plane of reflection).

For the types of symmetry involving a single rotation, the configuration of landmarks can be divided into sectors, which are the repeated units (Figure 4; these sectors can be viewed as a simplified version of the orbifold of this symmetry group [47]). To compute the symmetry and asymmetry components of the shape space, it is useful to distinguish the landmarks in the centre of rotation (or on the axis of rotation for 3D data) from the landmarks in each sector (Figure 4). If the order of rotation is denoted *o*, there are *o *sectors, each with *k *landmarks, and there are *c *landmarks on the centre or axis of rotation (*c *= 0 or 1 for 2D; *c *≥ 0 for 3D), so that the total number of landmarks is *ko *+ *c*.

For symmetry involving rotation only, shape variation has a symmetric and an asymmetric component (Table 2, upper part). For the symmetric component, the positions of landmarks in one sector can vary freely, which completely determines the landmark shifts in all other sectors; the central landmarks can shift only for 3D data, in the direction of the axis of rotation; and there are global constraints due to size and orientation and, for 3D data, due to position along the axis of rotation. For the asymmetric component, the landmarks of all sectors but one can move freely; the central landmarks can vary (perpendicular to the axis of rotation in 3D); and there are constraints due to overall position and, for 3D data, due to orientation (rotations around axes perpendicular to the axis of the rotation in the symmetry group). For rotations of order three or greater, the dimensionality of the asymmetric component is therefore substantially greater than that of the symmetric component. These degrees of freedom sum up to the total dimensionality of the shape tangent space, and the different components of variation occupy complementary subspaces in shape tangent space.

**Table 2:** Number of dimensions in the different components of shape space under object symmetry with rotation only or with rotation and reflection, for landmark data in two and three dimensions

	2D	3D
Symmetry under rotation only:		
Symmetric	2*k *- 2	3*k *+ *c *- 3
Asymmetric	2*k*(*o *- 1) + 2*c *- 2	3*k*(*o *- 1) + 2*c *- 4

Symmetry under rotation and reflection:		
Completely symmetric	2*p *+ *b *+ *m *- 1 = *k *- 1	3*p *+ 2*b *+ 2*m *+ *c *- 2
Reflection symmetry only	2*p*(*o *- 1) + *b*(*o *- 1) + *m*(*o *- 1) + *c *- 1 = *k*(*o *- 1) + *c *- 1	If *o *is even: $3p\left(o-1\right)+b\left(\frac{3}{2}o-1\right)+m\left(\frac{3}{2}o-2\right)+c-2$ If *o *is odd: $3p\left(o-1\right)+3b\frac{o-1}{2}+3m\frac{o-1}{2}+c-2$
Rotational symmetry only	2*p *+ *b *+ *m *- 1 = *k *- 1	3*p *+ *b *+ *m *- 1
Completely asymmetric	2*p*(*o *- 1) + *b*(*o *- 1) + *m*(*o *- 1) + *c *- 1 = *k*(*o *- 1) + *c *- 1	If *o *is even: $3p\left(o-1\right)+b\left(\frac{3}{2}o-2\right)+m\left(\frac{3}{2}o-1\right)+c-2$ If *o *is odd: $3p\left(o-1\right)+3b\frac{o-1}{2}+3m\frac{o-1}{2}+c-2$

Notation: For rotational symmetry of order *o*, the complete landmark configuration can be subdivided into *o *different sectors (Figure 4). Each sector contains *k *landmarks. In addition, there are *c *landmarks on the centre or axis of rotation (for 2D data, *c *is 0 or 1; for 3D data, *c *is 0 or greater). The sample consists of *n *individuals (specimens), and each specimen has been digitized *r *times.

If the symmetry group includes both rotation and reflection (with the reflection plane containing the rotation axis), there is a totally symmetric component of variation and the asymmetric component can be subdivided into a component symmetric under reflection only, a component symmetric under rotation only, and a component that is symmetric under neither rotation nor reflection (Table 2, lower part). For this type of symmetry, each sector of the landmark configuration is bilaterally symmetric and it is possible to distinguish three kinds of landmarks in each sector (Figure 4): there are *b *landmarks on each of the boundaries between sectors, *m *on the midline or mid-plane of each sector, and *p *pairs of corresponding landmarks on either side of the midline (*k *= 2*p *+ *b *+ *m*). For working out the degrees of freedom of each component (Table 2), the focus is on sectors for the rotational aspects of symmetry (as above) or on the paired and unpaired landmarks for aspects concerning reflection (as for bilateral symmetry [31,35]). For 3D data, it is necessary to distinguish cases with even and odd order of rotation because the plane of symmetry runs through two sector boundaries if the order of rotation is even and through a boundary and a mid-plane if the order of rotation is odd (Table 2). For all these cases, the degrees of freedom for the different components of variation sum up to the dimensionality of the shape tangent space and the components of variation define complementary subspaces.

For types of symmetry groups where the asymmetric component of shape variation can be subdivided into multiple complementary subspaces (e.g. the group of rotation and reflection, lower part of Table 2), this separation applies both to directional and fluctuating asymmetry. These components can be studied separately and may provide insight into the structure of morphological variation.

Because the symmetric and asymmetric components of shape variation are orthogonal subspaces of shape tangent space, they can also be identified and characterized through a principal component analysis (PCA) of the Procrustes-superimposed data in the combined sample of the original and transformed copies of landmark configurations. This approach can be used for any type of symmetry. It is a direct extension of the method for bilateral symmetry, where it can be shown that the symmetric and asymmetric components of variation in the combined sample do not covary [63]. Accordingly, the PCs are each unambiguously associated with a single component of the shape space, and the shape change associated with each PC shows the corresponding type of symmetry or asymmetry. The exception are analyses involving rotations of order 3 or greater, in which some pairs of PCs have equal eigenvalues, so that the direction of those PCs in the plane that they span together is not defined; accordingly, they can be rotated arbitrarily in that plane and the symmetry may not be visible in the pattern of shape changes associated with either of the PCs of the pair. An alternative procedure, which avoids this specific problem, is to perform PCA of the covariance matrices from the decomposition of variation by a Procrustes ANOVA.

### Procrustes ANOVA for complex symmetry

To quantify the different components of variation, we offer an extension to the Procrustes ANOVA for bilateral symmetry [29,31]. For bilateral symmetry, Procrustes ANOVA follows the design of the two-factor ANOVA proposed by Leamy [51] and Palmer and Strobeck [52] for linear measurements, which includes sides and individuals as main effects and their interaction. The main effect of individuals (variation among individual means, averaging across sides) represents individual variation, the main effect of sides (difference between sides, averaging asymmetries across individuals) represents directional asymmetry, and the individual-by-side interaction (the individual variation in asymmetry) represents fluctuating asymmetry [51,52]. This reasoning was adapted directly for shape analyses in the context of matching symmetry [29], whereas the reflection and relabelling was substituted for the factor of side in analyses of object symmetry [31]. The same design can be modified for more complex types of symmetry by replacing the factor of sides or reflection with the appropriate components of symmetry. Additional components of variation, such as replicate measurements to quantify measurement error, can easily be added [29,31,52].

#### Procrustes ANOVA for complex matching symmetry

For Procrustes ANOVAs of complex symmetries in the framework of matching symmetry, the units of analysis are the repeated parts (e.g. petals of flowers) on which landmarks are recorded. Landmark configurations for all parts of all individuals are included in the analysis. The simplest structure for the Procrustes ANOVA is then to include individuals and repeated parts as factors, and the main difference to the corresponding analysis for bilateral symmetry is that the factor of repeated parts may have more than two levels (e.g. five petals instead of two sides).

The interpretation of effects in the Procrustes ANOVA, in this case, closely follows that for bilateral symmetry. The main effect of individuals represents individual variation; because it is computed from averaging of all repeated parts in each individual, this component of variation is "symmetrized". The main effect of repeated parts represents directional asymmetry, the average asymmetry in the sample. Because of the complex symmetry, directional asymmetry is not just the difference between left and right averages, as for bilateral symmetry, but it represents the variation among the means of the different parts, averaged over all individuals. For the example of a flower with five petals, this means that directional asymmetry is represented by the shape differences of the five mean shapes of the petals from the overall mean shape. Finally, the individual × repeated-part effect represents fluctuating asymmetry, the individual variation in the differences among the repeated parts (just as fluctuating asymmetry, in the two-factor design for bilateral symmetry, is the individual variation in left-right differences [51,52]). Overall, therefore, the Procrustes ANOVA for bilateral symmetry can be extended to complex matching symmetry in a quite direct manner.

If there is a more complex structure in the arrangement of repeated parts, however, this can be accommodated by dividing the factor for repeated parts into multiple factors that capture aspects of the differences between repeated parts. We call these collectively "asymmetry factors". For instance, if four parts are arranged as quadrants (Figure 1A) and a dorsalventral axis can be distinguished from a left-right axis, then it is possible to include two asymmetry factors, one for each of the two axes, with two levels each. This is just a redistribution of levels from a single factor for repeated parts to two factors (and their combinations). Similar designs are also possible for matching symmetry of structures involving translational symmetry (e.g. for structures such as ribs, where serial homology is combined with left-right symmetry).

The interpretation of the effects in the Procrustes ANOVA is somewhat more intricate in this case because there are additional effects to be considered. As before, the main effect for individuals represents the variation among individuals and is symmetrized completely. The main effects of the asymmetry factors (e.g. dorsal-ventral and left-right) represent directional asymmetry in each particular aspect, symmetrized with respect to the other aspects (e.g. directional dorsal-ventral asymmetry, symmetrized for left-right differences, or directional left-right asymmetry, symmetrized for dorsal-ventral differences). This means that each of the aspects of symmetry is associated with a component of directional asymmetry that isolates this particular aspect and "averages out" other asymmetries. An additional component of directional asymmetry comes from the interaction between asymmetry factors, such as the interaction between the dorsal-ventral and the left-right effect. This component of directional asymmetry represents deviations of each part from the asymmetry expected from adding together the separate components of asymmetry (e.g. how the average shape of each quadrant differs from the asymmetry expected by adding the effects of the dorsal-ventral and the left-right asymmetries). Overall, directional asymmetry is divided into three components (or more, if there are more than two asymmetry factors). The interaction effects between the factor for individuals and the asymmetry factors represent components of fluctuating asymmetry. Again, there are several of these components: the two-way interactions between individuals and each of the asymmetry factors represent fluctuating asymmetry in just that aspect of asymmetry (e.g. in the dorsal-ventral or left-right components separately, symmetrized for the other aspects of asymmetry) and the three-way interaction between individuals and both asymmetry factors represents fluctuating asymmetry of each individual part as deviations from the added effects of the two asymmetry factors (deviations of each part from the asymmetry expected for that quadrant by adding the dorsal-ventral and left-right asymmetries). In summary, directional and fluctuating asymmetry are divided into components that represent particular aspects of asymmetry and can be used to address specific biological questions about the organisms under study.

Statistical testing in the context of Procrustes ANOVA for complex matching symmetries is similar to Procrustes ANOVA for bilateral matching symmetry [29,31]. Statistical inference can either be based on Goodall's *F *test [64] or a MANOVA approach [31]. Because all effects of the Procrustes ANOVA concern the same shape space, that of the entire landmark configuration of the repeated part, application of these statistical tests is fairly straightforward.

#### Procrustes ANOVA for complex object symmetry

As for matching symmetry, the Procrustes ANOVA for object symmetry with complex types of symmetry provides a similar decomposition of the variation into symmetric variation and one or more components of asymmetry. For any type of symmetry, it is possible to conduct Procrustes ANOVA with a single asymmetry factor that includes all transformations in the symmetry group as its levels. This follows the decomposition of the sum of squared Procrustes distances (equation 4).

Depending on the type of symmetry of the objects under study, it may be possible to use two or more transformations in the symmetry group as separate asymmetry factors in the ANOVA model. Specifically, the transformations used as asymmetry factors in the Procrustes ANOVA must be a set of generators of the symmetry group (a set of transformations that, under repeated application and in combination with each other, produce all the transformations in the symmetry group). Because some symmetry groups have multiple sets of generators, there may be an element of choice for the investigator. For instance, for the algal cell in Figure 1A, one set of generator consists of two reflections, one each about the horizontal and the vertical axis and the other set comprises a reflection (about either axis) and a rotation by 180° [39]. Although different choices of generator sets are equivalent in characterizing the symmetry group, some choices may have more obvious and intuitive biological interpretations than others (e.g. two reflections in dorsal-ventral and left-right directions may be more easily interpretable than a reflection and a rotation by 180°).

As in the Procrustes ANOVA for matching symmetry, the main effect of individuals represents among-individual variation; shape changes associated with this effect are completely symmetric because it is averaged over all transformed and relabelled copies of each individual configuration. The main effects of the asymmetry factors represent the respective components of directional asymmetry (e.g. average asymmetries with respect to rotations or reflections), each symmetrized with regard to the effects of all other asymmetry factors. The interaction effects between asymmetry factors characterize the components of directional asymmetry (those features of directional asymmetry that result from combinations of the different asymmetry factors). The interaction effects between the factor for individuals and the asymmetry factors represent the different components of fluctuating asymmetry (if more than one asymmetry factor is included, there are multiple components of fluctuating asymmetry).

Whether multiple asymmetry factors can be used in the Procrustes ANOVA depends on the type of symmetry. We only illustrate this in more detail for symmetries consisting of a rotation with or without reflection, which cover the vast majority of biological structures with complex symmetry. The components of variation of the effects of individuals, the one or more asymmetry factors and their interaction effects occupy different subspaces of the shape tangent space. The dimensionality of those subspaces (Table 2) is important for computing the degrees of freedom for the Procrustes ANOVA.

If the symmetry group includes only a rotation around a single point or axis, the Procrustes ANOVA contains this rotation as the only asymmetry factor (Table 3). Variation among individuals is computed from the averages of all the rotated and relabelled copies of each configuration, and this component of variation is therefore symmetric under rotation. Because rotation is the only asymmetry factor, directional and fluctuating asymmetry consist of a single component each, which both occupy the subspace of shape tangent space that is asymmetric under rotation. The main effect of rotation corresponds to directional asymmetry: the average of the differences among the sectors (or alternatively, the deviation of each sector in the average of the unrotated configurations from the symmetric consensus). Fluctuating asymmetry is the individual variation of the rotational asymmetries, and therefore corresponds to the rotation × individual interaction. If each specimen has been digitized more than once, it is also possible to assess measurement error, which affects the entire shape space (i.e. both the symmetric and asymmetric components).

**Table 3:** Degrees of freedom in the Procrustes ANOVA for object symmetry with rotation only in two and three dimensions

Effect	2D	3D
Individual	(*n *- 1) × (2*k *- 2)	(*n *- 1) × (3*k *+ *c *- 3)
Rotation	2*k*(*o *- 1) + 2*c *- 2	3*k*(*o *- 1) + 2*c *- 4
Rotation × individual	(*n *- 1) × (2*k*(*o *- 1) + 2*c *- 2)	(*n *- 1) × (3*k*(*o *- 1) + 2*c *- 4)
Measurement error	(*r *- 1) × *n *× (2*ko *+ 2*c *- 4)	(*r *- 1) × *n *× (3*ko *+ 3*c *- 7)

Notation: For rotational symmetry of order *o*, the complete landmark configuration can be subdivided into *o *different sectors (Figure 4). Each sector contains *k *landmarks. In addition, there are *c *landmarks on the centre or axis of rotation (for 2D data, *c *is 0 or 1; for 3D data, *c *is 0 or greater). The sample consists of *n *individuals (specimens), and each specimen has been digitized *r *times.

If the symmetry group includes rotation and reflection, the Procrustes ANOVA includes both of them as asymmetry factors, which leads to a more complex decomposition of the total variation (Table 4). The main effect of individuals, as above, represents the variation among specimens and is completely symmetric because it is computed from averages of all transformed and relabelled copies of each configuration. The main effects of rotation and of reflection and the rotation × reflection interaction represent different components of directional asymmetry (asymmetry under rotation only, under reflection only, or asymmetry under both rotation and reflection). The rotation × individual, reflection × individual and rotation × reflection × individual interactions are the corresponding components of fluctuating asymmetry. The components of directional and of fluctuating asymmetry occupy three separate subspaces of shape space (Table 2, lower part): the main effects of rotation and the rotation × individual interaction occupy the subspace that is symmetric under reflection only, the main effect of reflection and the reflection × individual interaction occupy the subspace that is symmetric under rotation only, and the rotation × reflection and rotation × reflection × individual interactions occupy the completely asymmetric subspace. Again, measurement error concerns the entire shape space (the symmetric and all asymmetric components).

**Table 4:** Degrees of freedom in the Procrustes ANOVA for object symmetry with rotation and reflection in two and three dimensions

Effect	2D	3D
Individual	(*n *- 1) × (2*p *+ *b *+ *m *- 1) = (*n *- 1) × (*k *- 1)	(*n *- 1) × (3*p *+ 2*b *+ 2*m *+ *c *- 2)
Rotation	2*p*(*o *- 1) + *b*(*o *- 1) + *m*(*o *- 1) + *c *- 1 = *k*(*o *- 1) + *c *- 1	If *o *is even: $3p\left(o-1\right)+b\left(\frac{3}{2}o-1\right)+m\left(\frac{3}{2}o-2\right)+c-2$ If *o *is odd: $3p\left(o-1\right)+3b\frac{o-1}{2}+3m\frac{o-1}{2}+c-2$
Reflection	2*p *+ *b *+ *m *- 1 = *k *- 1	3*p *+ *b *+ *m *- 1
Rotation × reflection	2*p*(*o *- 1) + *b*(*o *- 1) + *m*(*o *- 1) + *c *- 1 = *k*(*o *- 1) + *c *- 1	If *o *is even: $3p\left(o-1\right)+b\left(\frac{3}{2}o-2\right)+m\left(\frac{3}{2}o-1\right)+c-2$ If *o *is odd: $3p\left(o-1\right)+3b\frac{o-1}{2}+3m\frac{o-1}{2}+c-2$
Rotation × individual	(*n *- 1) × (2*p*(*o *- 1) + *b*(*o *- 1) + *m*(*o *- 1) + *c *- 1) = (*n *- 1) × *k*(*o *- 1) + *c *- 1	If *o *is even: $\left(n-1\right)\times \left(3p\left(o-1\right)+b\left(\frac{3}{2}o-1\right)+m\left(\frac{3}{2}o-2\right)+c-2\right)$ If *o *is odd: $\left(n-1\right)\times \left(3p\left(o-1\right)+3b\frac{o-1}{2}+3m\frac{o-1}{2}+c-2\right)$
Reflection × individual	(*n *- 1) × (2*p *+ *b *+ *m *- 1) = (*n *- 1) × (*k *- 1)	(*n *- 1) × (3*p *+ *b *+ *m *- 1)
Rotation × reflection × individual	(*n *- 1) × (2*p*(*o *- 1) + *b*(*o *- 1) + *m*(*o *- 1) + *c *- 1) = (*n *- 1) × *k*(*o *- 1) + *c *- 1	If *o *is even: $\left(n-1\right)\times \left(3p\left(o-1\right)+b\left(\frac{3}{2}o-2\right)+m\left(\frac{3}{2}o-1\right)+c-2\right)$ If *o *is odd: $\left(n-1\right)\times \left(3p\left(o-1\right)+3b\frac{o-1}{2}+3m\frac{o-1}{2}+c-2\right)$
Measurement error	(*r *- 1) × *n *× (4*po *+ 2*bo *+ 2*mo *+ 2 *c *- 4) = (*r *- 1) × *n *× (2*ko *+ 2 *c *- 4)	(*r *- 1) × *n *× (6*po *+ 3*bo *+ 3*mo *+ 3 *c *-7)

Notation: For rotational symmetry of order *o *and reflection, the complete landmark configuration can be subdivided into *o *different sectors. Because of the reflection symmetry, each sector must be symmetric under reflection. Therefore, the *k *landmarks contained in each sector can be subdivided into *b *landmarks on the boundary between sectors, *m *landmarks on the midline of the sector (the mid-plane for 3D data), and *p *pairs of corresponding landmarks on either side of the midline of the sector, so that *k *= 2*p *+ *b *+ *m*. The difference between boundaries and midlines of the sectors is important if the order of the rotation is even: in this case, the plane of reflection symmetry is assumed to pass through two boundaries between sectors (e.g. the vertical axis in Figure 4). In addition, there are *c *landmarks on the centre or axis of rotation (for 2D data, *c *is 0 or 1; for 3D data, *c *is 0 or greater). The sample consists of *n *individuals (specimens), and each specimen has been digitized *r *times.

Testing in the framework of Procrustes ANOVA for complex symmetries is similar to Procrustes ANOVA for bilateral symmetry [29,31], and can be based either on an extension of Goodall's F test [64] to the more complex ANOVA model or use a MANOVA approach [31]. The computations of the Procrustes sums of squares follow the same principles as for bilateral object symmetry, but care should be taken because there may be multiple asymmetry effects, each associated with a different subspace of the total shape space. In particular, this aggravates the problem how one should choose the error term for testing the effect of individuals (i.e. the completely symmetric component of variation) when the interaction effects are localized in different (asymmetric) subspaces [31]. Following the argument that the symmetric variation among individuals should be assessed against the fluctuating asymmetry within individuals [52], it is possible to add the Procrustes sums of squares and degrees of freedom for all the fluctuating asymmetry components (the rotation × individual, reflection × individual and rotation × reflection × individual interactions) to compute a pooled estimate of fluctuating asymmetry.

Finally, a note of caution about terminology may be useful. To count as symmetric under rotation for the Procrustes ANOVA, a shape change must be symmetric under rotation of the full order. But these may not be the only components of variation that are rotationally symmetric if the order of rotation is not a prime number. For instance, analyses involving rotation of order 6 may include shape features that are symmetric under rotations of order 2 or 3; these will be counted as shape changes that are asymmetric under rotation, because they are not symmetric under rotation of order 6. Likewise, reflections are defined in relation to a particular axis or plane of reflection; shape changes that are symmetric under reflection about a different axis or plane will therefore not be considered as symmetric under reflection for the purposes of the Procrustes ANOVA. Because these partial symmetries concern one or more of the components of fluctuating asymmetry, a PCA of the covariance matrices for the respective effects can reveal this additional structure in the data. The order of rotation and axis or plane of reflection should be chosen to relate to the anatomical features and the biological question under investigation. All these considerations are best illustrated by an example.

### Case study: Shape analysis of symmetry and asymmetry in a colonial coral

**Figure 5:**
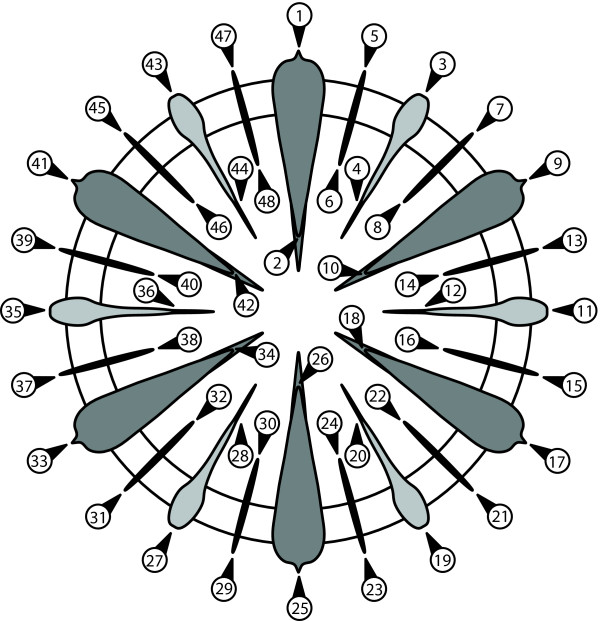
**Schematic representation of a corallite with the landmarks used in this study**. The septa colored in dark grey belong to the first cycle, the ones in light grey to the second cycle, and those in black to the third cycle. The ring in white represents the mural structure of the corallite.

Our case study concerns the symmetry and asymmetry of corallites in a colonial coral (*Galaxea sp*.). In this coral, the corallites do not abut directly, but are separated from each other by a small distance. Corallites possess radially projecting partitions called septa, which are prominent in the apical view of each corallite (Figure 5). The arrangement of septa is consistent with several types of symmetry: rotational symmetry of order 2, 3, or 6, either with or without reflection symmetry. Because of this ambiguous type of symmetry, corallites are a somewhat unusual and particularly suitable example that allows us to demonstrate different types of symmetry in a single structure. We consider the corallites as structures that are symmetric as a whole, and therefore apply the method for analyzing structures with object symmetry and different symmetry groups. We illustrate our approach by analyzing three different types of symmetry: (1) reflection symmetry combined with rotational symmetry of order 2 (biradial symmetry or disymmetry), (2) rotational symmetry of order 6, and (3) reflection symmetry combined with rotational symmetry of order 6.

We characterize the shape of 50 corallites from a single colony by a configuration of 48 landmarks (Figure 5), so that the shape tangent space has 92 dimensions (48 landmarks × 2 - 4). None of the landmarks is in the centre of rotation, so that *c *= 0, and there are 24 or 8 landmarks per sector for analyses with rotations of order 2 and 6, respectively (cf. Figure 4 and Tables 1, 2, 3). Two images of each corallite were taken and each image was digitized twice.

In all analyses, we use a full Procrustes fit of all transformed and relabelled copies of the landmark configurations and the data are projected into the tangent space by orthogonal projection [49]. For each of these analyses, we conduct a Procrustes ANOVA with the appropriate design (Tables 2, 3). Because the main aim of these analyses is to illustrate how different types of symmetry partition the total variation into components of symmetric and asymmetric variation, only the method of Goodall's *F *[64] is used for statistical testing.

In addition, principal component analysis (PCA) is also used to separate different components of symmetric and asymmetric shape variation and to display the corresponding shape changes [31,63]. This approach differs from Procrustes ANOVA in that it considers the total variation around the completely symmetric mean shape without distinguishing sources of variation for each subspace (e.g. without separating directional and fluctuating asymmetry or measurement error). We use PCA to separate the various components of shape variation in our different example analyses. The symmetric and asymmetric components of shape variation that can be extracted from the PCA depend on the symmetry transformations included in the dataset. When rotations of order 3 or greater are included in the symmetry group (in analyses 2 and 3), the PCA yields a series of pairs of PCs with equal eigenvalues. For each of these pairs, the PCs are not uniquely defined, and any pair of perpendicular directions in the corresponding plane can be chosen as the PCs. We therefore rotate the PCs of these pairs so that the deviation of the mean score of all unrotated copies from the overall mean is aligned with the first of the PCs in the pair. As a result, the shape changes associated with the PCs fall into recognizable types. In each analysis, we describe one PC for each category of shape variation as an example.

#### Analysis 1: Reflection and rotation of order 2

The first analysis uses the type of symmetry with two perpendicular axes of reflection symmetry, also known as biradial symmetry or disymmetry (e.g. Figure 1A). The symmetry group contains four symmetry transformations: the identity, a reflection about the vertical axis, rotation by 180° (which is equivalent to two successive reflections about the vertical and horizontal axes), and a combination between reflection and rotation by 180° (which is equivalent to a reflection about the horizontal axis).

The shape tangent space consists of four subspaces: a component that is symmetric under both rotation and reflection (about both horizontal and vertical axes), a component symmetric under rotation by 180° (but not under any reflection), a component symmetric under reflection about the vertical axis and, finally, a component that is not symmetric under either rotation or reflection about the vertical axis (but due to the constraints imposed by the Procrustes fit, it is symmetric under reflection about the horizontal axis). Each of these subspaces has 23 dimensions (Table 2).

The Procrustes ANOVA includes individuals, rotation by 180° and reflection about the vertical axis as the factors (Table 5). A comparison of the mean squares (Table 5) indicates that the reflection effect appears to be the largest of the components of directional asymmetry and reflection × individual the largest component of fluctuating asymmetry (but the main effect of reflection is not statistically significant if tested against the reflection × individual interaction). Both concern the deviations from bilateral symmetry about the vertical axis that are symmetric under rotation by 180°, such as compression or stretching in an oblique direction.

**Table 5:** Procrustes ANOVA for the coral example, with a symmetry group consisting of reflection and rotation of order 2

Source	Degrees of freedom	Sums of squares	Mean squares	*F*	*P*
Individual	1127	4.9380	0.0043815	1.82	< 0.000001
Rotation	23	0.096360	0.0041896	4.20	< 0.000001
Reflection	23	0.16058	0.0069818	1.34	0.13
Rotation × reflection	23	0.072451	0.0031501	3.17	< 0.000001
Rotation × individual	1127	1.1251	0.0009983	4.95	< 0.000001
Reflection × individual	1127	5.8839	0.0052209	25.87	< 0.000001
Rotation × reflection × individual	1127	1.1184	0.0009924	4.92	< 0.000001
[Total FA]	3381	8.1274	0.0024039		
Imaging error	4600	0.92842	0.0002018	1.26	< 0.000001
Digitizing error	9200	1.4789	0.0001607		

The main effect of individuals is tested against the mean square for the total fluctuating asymmetry ("Total FA": pooling sums of squares and degrees of freedom across all three subspaces with asymmetric variation: rotation × individual, reflection × individual and rotation × reflection × individual). This total asymmetry is not used otherwise in the analysis.

**Figure 6:**
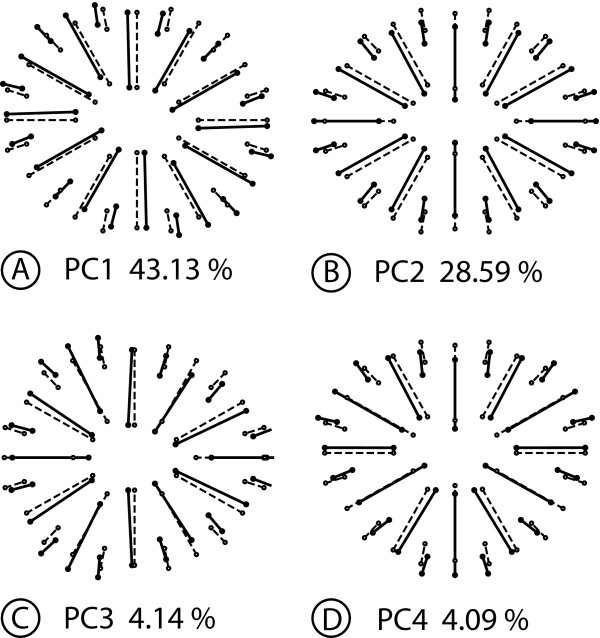
**Analysis 1: Decomposition of shape variation for symmetry with respect to reflection and rotation of order 2**. This figure shows examples of PCs that account for the maximum of variance for each category of shape variation. Each diagram shows the symmetric consensus (open circles and dotted lines) and the differences between the consensus and the other configuration (solid circles and solid lines) represent the shape change associated with the respective PC by an arbitrary amount of + 0.1 units of Procrustes distance. The percentages represent the part of the total shape variation for which each PC accounts. A. Asymmetric component, symmetric under rotation of order 2. B. Symmetric component. C. Asymmetric component, symmetric relative to reflection about the horizontal axis. D. Asymmetric component, symmetric under reflection about the vertical axis.

In accordance with the structure of the shape space, the PCA produces PCs associated with four types of shape changes that differ in their symmetries. The PC1 is symmetric under rotations of order 2, but not under reflection (Figure 6A). The shape change is an expansion and compression in oblique directions, so that the corallites change from the roughly circular cross-section in the consensus to an elliptic shape with the long axis oriented either from upper-left to lower right (positive PC1 score, Figure 6A) or from lower-left to upper-right (negative score, not shown). The shape change of the PC2 is completely symmetric (Figure 6B) and primarily shows variation in the relative expansion and contraction along the horizontal and vertical axes. The PC3 is symmetric under reflection about the horizontal axis (asymmetric under both rotation and reflection about the vertical axis) and includes a deformation of the overall contour of the corallite into a more triangular shape pointing either to the left or right side (Figure 6C). Finally, the PC4 represents a fourth type of shape change, which is symmetric under reflection about the vertical axis, as there are mirror-image changes on the left and right sides (Figure 6D).

Because each PC can be unambiguously allocated to one of the components of shape space, it is possible to count the dimensions in the respective subspaces and to add up the corresponding eigenvalues to quantify the amount of total variance for each type of symmetry (note that this also includes the imaging and digitizing errors, which are not separated according to components by the Procrustes ANOVA). The 23 PCs that are symmetric under rotation by 180° account for nearly half the total shape variation (45.48% of the total variance). The 23 completely symmetric PCs account for 35.38% of the total variance, 9.09% of the total variance are apportioned to the 23 PCs that are symmetric under reflection about the horizontal axis (asymmetric under both rotation and reflection about the vertical axis), and 9.05% of the total variance are allocated to the 23 PCs that are symmetric with respect to reflection about the vertical axis. These numbers of PCs correspond to the numbers of dimensions in the respective subspaces.

#### Analysis 2: Rotation of order 6

For the analysis of symmetry under rotation of order 6, the symmetry group contains six symmetry transformations: rotations by 60°, 120°, 180°, 240°, 300°, and 360° (the latter is the same as the identity).

The analysis partitions the shape tangent space into a 14-dimensional symmetric component and a 78-dimensional component that is not symmetric under rotations of order 6. In the Procrustes ANOVA, the main effect of rotation and the rotation × individual interaction dominate both the sums of squares and mean squares (Table 6). This indicates that most of the variation is not symmetric under rotation of order 6.

**Table 6:** Procrustes ANOVA for the coral example, with a symmetry group consisting of rotation of order 6

Source	Degrees of freedom	Sums of squares	Mean squares	*F*	*P*
Individual	686	0.79714	0.001162	0.24	> 0.999999
Rotation	78	7.6145	0.097622	20.04	< 0.000001
Rotation × individual	3822	18.616	0.0048708	16.08	< 0.000001
Imaging error	4600	1.3934	0.0003029	1.25	< 0.000001
Digitizing error	9200	2.2213	0.0002414		

**Figure 7:**
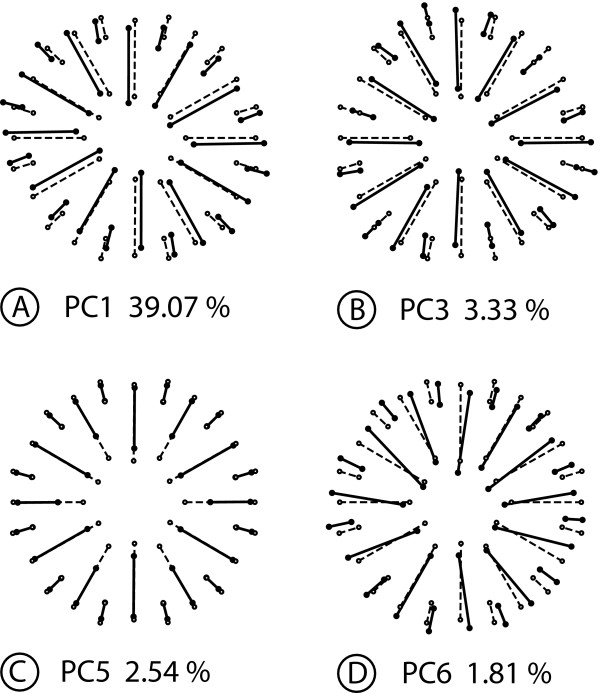
**Analysis 2: Decomposition of shape variation for symmetry with respect to rotation of order 6**. This figure shows examples of PCs that account for the maximum of variance for each category of shape variation. Each diagram shows the symmetric consensus (open circles and dotted lines) and the differences between the consensus and the other configuration (solid circles and solid lines) represent the shape change associated with the respective PC by an arbitrary amount of + 0.1 units of Procrustes distance. The percentages represent the part of the total shape variation for which each PC accounts. A. Asymmetric component, symmetric under rotation of order 2. B. Asymmetric component, symmetric under rotation of order 3. C. Symmetric component. D. Totally asymmetric component.

The 92 PCs are divided into four types of shape changes. The first category includes the PC1, which is symmetric under rotations of order 2 (Figure 7A). The corresponding shape change consists of a combination of a deformation of the overall contour of the corallite and relative shifts of the septa similar to that seen in the previous analysis (Figure 6A). The PC3 represents the second type of shape changes, which is symmetric under rotation of order 3 (Figure 7B). This shape change combines rotational shifts of the septa and variation between a more circular and a more triangular cross-section of the corallite. The third type is symmetric under rotation of order 6, and therefore constitutes the symmetric component of variation. This type of shape change includes the PC5 (Figure 7C), which consists of landmark shifts in almost precisely radial directions (this is a particular feature of this PC, and other PCs show various clockwise or counter-clockwise shifts of landmarks). Finally, the PC6 represents the last type of shape change, which is totally asymmetric and shows irregular shifts of all landmarks (Figure 7D).

In total, 32 PCs are symmetric under rotation of order 2 and account for most of the variance (82.63% of the total variance), 16 are symmetric under rotation of order 3 (7.15% of the total variance), 14 are symmetric under rotation of order 6 (the symmetric component of the Procrustes ANOVA) and represent 3.43% of the total variance, and 30 are completely asymmetric (6.79% of the total variance). Above all, it is notable that the PCA further subdivides the 78 dimensions of the asymmetric component of variation of the Procrustes ANOVA.

#### Analysis 3: Reflection and rotation of order 6

For the analysis of symmetry under reflection and rotation of order 6, the symmetry group includes twelve symmetry transformations: each of the six rotations of the preceding analysis is now included with or without reflection.

There are four subspaces: a 7-dimensional subspace of completely symmetric shape changes, a 7-dimensional subspace of shape changes that are symmetric under rotations of order 6 but not under reflection, a 39-dimensional subspace of shape changes that are symmetric under reflection about the vertical axis but not under rotation of order 6, and a 39-dimensional shape space of completely asymmetric shape changes. The Procrustes ANOVA shows that, for both the sums of squares and mean squares (Table 7), the bulk of the variation is associated with effects involving rotation (i.e. shape changes that are not symmetric under rotation of order 6), both for directional asymmetry (the rotation and rotation × reflection effects) and for fluctuating asymmetry (the rotation × individual and rotation × reflection × individual effects).

**Table 7:** Procrustes ANOVA for the coral example, with a symmetry group consisting of reflection and rotation of order 6

Source	Degrees of freedom	Sums of squares	Mean squares	*F*	*P*
Individual	343	1.510	0.0044038	0.49	> 0.999999
Rotation	39	7.6968	0.19735	20.31	< 0.000001
Reflection	7	0.008013	0.0011447	4.69	0.000049
Rotation × reflection	39	7.5323	0.19314	19.78	< 0.000001
Rotation × individual	1911	18.569	0.0097171	16.04	< 0.000001
Reflection × individual	343	0.083800	0.0002443	0.40	> 0.999999
Rotation × reflection × individual	1911	18.663	0.009766	16.11	< 0.000001
[Total FA]	4165	37.316	0.0089594		
Imaging error	4600	2.7869	0.0006058	1.25	< 0.000001
Digitizing error	9200	4.4426	0.0004829		

The main effect of individuals is tested against the mean square for the total fluctuating asymmetry ("Total FA": pooling sums of squares and degrees of freedom across all three subspaces with asymmetric variation: rotation × individual, reflection × individual and rotation × reflection × individual). This total asymmetry is not used otherwise in the analysis.

**Figure 8:**
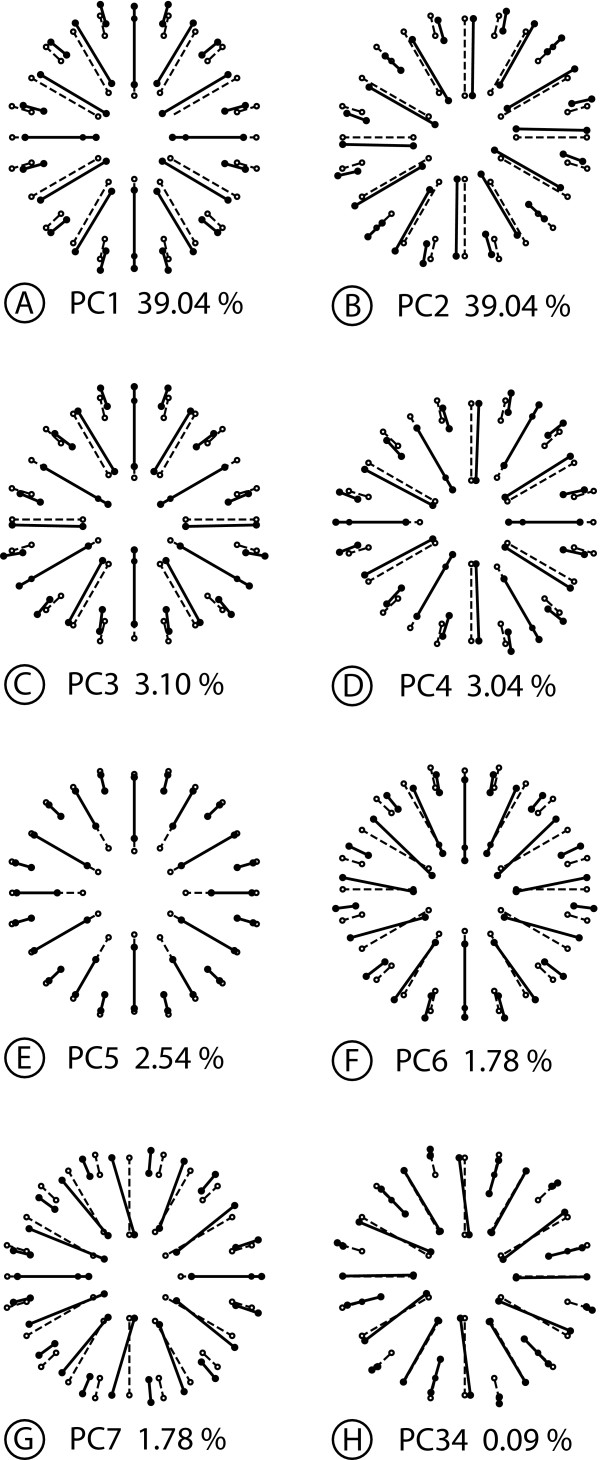
**Analysis 3: Decomposition of shape variation for symmetry under reflection and rotation of order 6**. This figure shows examples of PCs that account for the maximum of variance for each category of shape variation. Each diagram shows the symmetric consensus (open circles and dotted lines) and the differences between the consensus and the other configuration (solid circles and solid lines) represent the shape change associated with the respective PC by an arbitrary amount of + 0.1 units of Procrustes distance. The percentages represent the part of the total shape variation for which each PC accounts. A. Asymmetric component, symmetric under reflection and rotation of order 2. B. Asymmetric component, symmetric under rotation of order 2 but not reflection. C. Asymmetric component, symmetric under reflection about the vertical axis and rotation of order 3. D. Asymmetric component, symmetric under reflection about the horizontal axis and rotation of order 3. E. Completely symmetric component. F. Asymmetric component, symmetric under reflection about the vertical axis. G. Asymmetric component, symmetric under reflection about the horizontal axis. H. Asymmetric component, symmetric under rotation of order 6.

The PCs fall into eight distinct categories of shape changes (Figure 8). The PC1 represents the first type of shape change, which is symmetric under reflection and rotation of order 2 (Figure 8A). The PC2 stands for the second group of shape changes, which is symmetric under rotation of order 2 but not under reflection (Figure 8B). The shape variation associated with PC3 is symmetric under rotation of order 3 and reflection about the vertical axis (Figure 8C). The shape change of PC4 is also symmetric under rotation of order 3, but its reflection symmetry is about the horizontal and not the vertical axis (Figure 8D). The PC5 features a shape change that is totally symmetric under reflection and rotations of order 6 (Figure 8E; for this type of change, the shifts of landmarks on the sector boundaries and midlines are limited to the radial direction, but it is a coincidence that the remaining landmark shifts are also in a radial direction for this particular PC). The PC6 represents shape variation that is symmetric under reflection about the vertical axis (Figure 8F). The shape change associated with PC7 is symmetric under reflection about the horizontal axis (Figure 8G). The last type of shape changes, which is symmetric under rotations of order 6, includes the PC34 (Figure 8H).

Overall, patterns of shape variation symmetric under rotations of order 2 dominate the variation: 16 PCs are symmetric under rotation of order 2 and account for 41.3% of the total variance and 16 PCs are symmetric under both reflection and rotations of order 2 and account for a further 41.3% of the total variance. In addition, there are 15 PCs that are symmetric under reflection about the vertical axis and account for 3.4% of the total variance, and another 15 PCs are symmetric under reflection about the horizontal axis and also account for 3.4% of the total variance. There are 8 PCs that are symmetric under rotation of order 3 and reflection about the vertical axis and take up 3.5% of the total variance, whereas 8 PCs are symmetric under rotation of order 3 and reflection about the horizontal axis and account for 3.6% of the total variance. Finally, 7 PCs are completely symmetric and comprise 3.3% of the total variance, whereas 7 PCs are symmetric under rotation of order 6 and make up the remaining 0.2% of the total variance.

## Discussion

In this paper, we have introduced a new and general framework for shape analysis of symmetric and asymmetric variation in a configuration of landmarks with any type of symmetry. We have presented methods that implement this framework and extend the methods widely used for analyzing shapes with bilateral symmetry [28,29,31,35,36,65]. We have illustrated the new framework with a small case study on the shape variation of corallites in a coral colony.

In our case study, the Procrustes ANOVAs and PCAs for the three analyses produce different decompositions of the shape variation into components of symmetric and asymmetric shape variation. A common feature, however, is that the majority of the shape variation is contained in the asymmetric component. In the first analysis, with symmetry under reflection and rotation of order 2, the PCA indicates that asymmetric variation accounts for 64.62% of the total variance. In the two other analyses, which include rotation of order 6, the components of asymmetric variation take up more than 96% of the total variance in the PCA. The large proportion of shape variation that is taken up by the asymmetric component of variation is in striking contrast to most studies of shape variation in structures with bilateral symmetry [e.g. [14,25,29,31-33,53,66]]. This difference may relate to the fact that our analysis considers variation of sessile organisms that have grown in a heterogeneous environment. Corals are known to have a considerable degree of phenotypic plasticity [67-72]. The large proportion of asymmetric shape variation in all our analyses may therefore relate to the effects of directed environmental factors such as light and water movement, which have been shown to influence coral growth [72-75]. Effects of crowding by neighbouring corallites might add further asymmetric variation.

The PCAs reveal much more shape variation that is structured according to biradial symmetry (rotation of order 2 and reflection) than there is variation with hexagonal symmetry (rotation of order 6 and reflection). The components that are symmetric under rotation by 180° are consistently among the dominant PCs and account for about 40% of the total variance of shape (Figure 6A, 7B). Moreover, in the analyses including reflection, shape changes symmetric under reflection and rotation by 180° also take up a large share of the total shape variation (Figure 6B, 7A). The Procrustes ANOVAs suggest that these patterns apply to both directional and fluctuating asymmetry (Tables 3, 4, 5). These results reflect the fact that the cross-sections of most corallites in the sample are more or less oval or elliptic and not circular or hexagonal [see also [76]]. Accordingly, variation in the relative lengths and the orientation of the major and minor axes is a dominant feature of shape variation. Depending on the type of symmetry considered in a specific analysis, these shape changes appear as part of the overall component of symmetry or asymmetry. In contrast, only a small proportion of the variation is symmetric under rotation of order 6, which might be expected to predominate if corallite organization were to reflect the theoretical optimum of hexagonal packing of polyps on the colony surface. In *Galaxea*, unlike some other colonial corals, corallites are set apart from each other by a small distance, so that the dense packing of corallites may not be a primary factor determining patterns of variation.

For our example analyses, only a fairly small proportion of shape variation is symmetric. Some patterns of symmetric variation among corallites might be due to differences in septal growth. The septa develop from the mural structure of the corallite towards the centre and also towards the outside of the corallite [76]. The septa are arranged in four complete cycles, but only the first three are fully visible: six septa appear throughout the first, six septa develop during the second cycle and twelve septa grow during the third cycle (Figure 5). Changes in the relative development of the three first cycles have been captured by the completely symmetric PCs in the analyses including rotation of order 6 (Figure 7C, 8E, G). In the PC5 of both analyses, the shape changes correspond to radial shifts of landmarks along the septa that are identical for all septa that belong to the same developmental cycle. Such biological interpretations, however, should be made with caution because this study is based on a fairly small number of corallites from a single colony and because the development of corallites is complex and incompletely known [e.g. [76,77]].

Although the Procrustes ANOVAs and the PCAs both produce broadly similar results, these analyses differ substantially in how they partition the total variation into components. The Procrustes ANOVA allocates shape variation to various components that reflect the study design and data collection, such as directional and fluctuating asymmetry or imaging and digitizing error, even if several of these components are located in the same subspace. The components extracted by the Procrustes ANOVA, however, may be somewhat heterogeneous in their symmetries (e.g. the component of variation considered asymmetric under rotation of order 6 may contain shape changes that are symmetric under rotations of orders 2 or 3 or under reflections). In contrast, the PCA separates components according to the structure of the shape tangent space, and can therefore identify more classes of shape changes according to their symmetries. The PCA, however, does not consider other aspects of the data structure--importantly, it provides only a total characterization of asymmetry that does not distinguish between directional and fluctuating asymmetry. The PCA considers asymmetry as the total of the deviations from complete symmetry. PCA does not distinguish whether variation stems from consistent differences between repeated parts that are shared among individuals and are therefore directional asymmetry, or whether variation reflects individual differences in the deviations from symmetry and is therefore fluctuating asymmetry. Overall, therefore, Procrustes ANOVA and PCA give somewhat different perspectives on the variation in the data, which is most important for the interpretation of asymmetry.

The three analyses, based on different symmetry groups, produce different estimates of the consensus shape and different partitions of the observed shape variation into components with distinct types of symmetry. The number of these components is influenced primarily by the number of symmetry transformations for the type of symmetry considered and, accordingly, by the number of transformed copies of each landmark configuration that are included in the dataset. In the first analysis, only four transformed copies are included in the dataset, whereas six transformed copies are considered in the second analysis, and twelve transformed copies are included in the third analysis. More complex types of symmetry are associated with symmetry groups that consist of greater numbers of transformations, and thus produce more types of PCs.

These differences between analyses of the same data raise the question how the most appropriate type of symmetry should be chosen. The coral example was included specifically because of its ambiguous symmetry, so that several types of symmetry can be demonstrated with the same data, and this analysis should therefore not be viewed as a model for studies of complex symmetries in general [for such an example, see [39]]. Normally, the type of symmetry should be chosen according to criteria that are relevant to the biological context of a particular study, and will most often consider anatomical and developmental criteria. For most analyses, the choice will be much more straightforward than for the example in this study, because the number and arrangement of repeated parts is unambiguous (e.g. the number of petals in a flower). Also, for the coral example, there is biological information that clearly favours some types of symmetry over others. In corals, skeletal structures may have hexagonal, biradial or bilateral symmetry depending on the taxonomic group, but soft parts tend to have either biradial or bilateral symmetry [e.g. [78-83]]. Developmental studies in the sea anemone *Nematostella vectensis *have shown that many genes involved in the organization of the adult body plan are expressed in bilaterally symmetric patterns [e.g. [5,6]]. Overall, therefore, these biological considerations suggest that biradial or bilateral symmetry is more appropriate for studying corals than the types of symmetry involving a rotation of order 6. With a choice of symmetry group that is appropriate for the organisms under study, methods introduced and demonstrated in this paper can be used to extract biologically interesting features from the different components of symmetric and asymmetric variation [39].

### Alternative approaches

A few different morphometric methods for the analysis of complex symmetries have been suggested in recent years [37,38]. Frey et al. [37] proposed an approach for the analysis of rotational symmetry. If a landmark configuration is rotationally symmetric, then each set of corresponding landmarks forms a perfect polygon. The method uses linear measurements and angles among landmarks to characterize the polygon formed by the actual landmarks and computes a measure of its deviation from a perfect polygon. Frey et al. [37] used this method to examine how well the tips of the petals of flowers with five petals corresponded to perfect pentagons and found that the method performed well in ranking more or less symmetric flowers. The drawback of this framework is that it is limited to the study of rotational symmetry and to only a single landmark per repeated sector. Our approach is more general because it is not limited to a specific number of landmarks per sector or to a type of symmetry.

Potapova and Hamilton [38] extended the method for analyzing bilateral object symmetry for the analysis of diatoms that are symmetric with respect to two perpendicular axes of symmetry (biradial symmetry). They used an original configuration of landmarks for each entire diatom, generated three copies to which they applied reflections about the two axes of symmetry, and appropriately relabelled the landmarks of each transformed copy. Although Potapova and Hamilton [38] did not describe it in these terms, their analysis applied the four symmetry transformations that define the full symmetry group for biradial symmetry. Therefore, Potapova and Hamilton's approach is identical to the method used here for that type of symmetry (Analysis 1), and it is a special case of the more general framework we presented in this study. Savriama et al. [39] applied the current framework for analyzing biradial symmetry in algal cells of *Micrasterias rotata *to extract symmetric and asymmetric components of variation and related them to the processes of cellular growth.

Finally, the approach of Zabrodsky et al. [40,41,84] is particularly intriguing because of its relationships with the current methodology. Their work was developed in the context of chemistry and computer vision, independently from similar work in morphometrics and statistical shape analysis. Zabrodsky et al. [41] defined a symmetry distance that quantifies how much a set of points deviates from a corresponding, perfectly symmetric shape. Like the Procrustes approach, this method is based on minimizing the sum of squared distances between corresponding points. Up to a scaling factor, the symmetry distance is identical to the squared Procrustes distance between the original landmark configuration and the completely symmetric consensus of our approach. The approach is suitable for object symmetry of any type, that is, for any finite symmetry group. The main difference is that the approach of Zabrodsky et al. [41] is for assessing how symmetric a single shape is, and that it therefore is not equipped to analyze the variation among several configurations in symmetric aspects of shape or in asymmetries. Graham et al. [12] point out that, in the absence of directional asymmetry, the symmetry distance can be used as a measure of fluctuating asymmetry; this is equivalent to the use of Procrustes distance [29,85] (up to squaring and as long as total shape variation is small, so that the differences between methods for size scaling result only in a difference by a scaling factor that is approximately constant). Because the Procrustes approach can estimate and correct for directional asymmetry, it has the advantage that it can be used as a measure of fluctuating asymmetry even if directional asymmetry is present [29,85].

## Conclusion

The approach we have introduced in this paper provides powerful morphometric tools for biologists to analyze symmetry and asymmetry in landmark data. The method generalizes the approach previously used for bilateral symmetry for studies of landmark configurations with any possible type of symmetry and provides a unified perspective on biological symmetry. Previous insights about the structure of the shape tangent space for bilateral symmetry [35,36,63] also can be extended in this much more general setting. Similarly, we have extended the Procrustes ANOVA for quantifying the different components of shape variation [29,31] for any type of symmetry. Other morphometric tools can be used in this new context as well.

The use of complex asymmetries, where more than two repeated parts can be compared, avoids some of the problems of fluctuating asymmetry in organisms with bilateral symmetry [86,87]. For instance, analyses of radially symmetric flowers can measure the lengths of multiple petals [88,89] or the arrangement of all the petal tips [37]. Because the approach outlined in this paper considers multiple landmarks per iterated part, it makes more information available for such studies and is therefore likely to provide greater power and sensitivity. This applies both to studies that use fluctuating asymmetry as a measure of developmental instability and to those using it in analyses of morphological integration. Because the complex symmetry of a structure often relates to its development and growth, for instance through the arrangements of septae of corallites (Figure 5) or the structure of algal cells [39], the patterns for different components of symmetric and asymmetric variation may provide a more detailed and multifaceted picture than it is possible for bilateral symmetry. For these reasons, we think that our framework for the analysis of complex symmetries is a useful tool for studies of the evolution and development of the shapes of biological structures [11].

## Methods

Our case study concerns the symmetry and asymmetry of corallites in a specimen of colonial coral (*Galaxea sp*.) from the collection of the Manchester Museum. Digital photographs of 50 corallites from a single colony were taken in apical view with a Leica M420 macroscope and attached digital camera. Two images of each corallite were taken in separate sessions (i.e. the colony was positioned under the microscope separately for each image). For each picture, 48 landmarks were digitized (Figure 5) using tpsDig, version 2.05 [90]. The landmarks were digitized twice on all images. Analyses were carried out using SAS/IML [91] and MorphoJ [92].

## Competing interests

The authors declare that they have no competing interests.

## Authors' contributions

YS and CPK jointly devised the methodology. YS collected the data and conducted the analyses for the case study. YS and CPK wrote the paper. Both authors read and approved the final manuscript.
